# Multiple paragangliomas of head and neck associated with hepatic paraganglioma: a case report

**DOI:** 10.1186/s12880-015-0082-z

**Published:** 2015-09-25

**Authors:** Zebin Xiao, Dejun She, Dairong Cao

**Affiliations:** Department of Radiology, First Affiliated Hospital of Fujian Medical University, 20 Cha-Zhong Road, Fuzhou, Fujian 350005 P.R. China

**Keywords:** Paraganglioma, Head and neck, Liver, Immunohistochemistry, CT, MR

## Abstract

**Background:**

Paragangliomas (PGs) are neuroendocrine tumors derived embryonically from the neural crest cells of the autonomic nervous system. Approximately 3 % of all paragangliomas occur in the head and neck area. Head and neck paragangliomas (HNPGs) are rare and highly vascularized tumors, the majority of which are benign. Multiple HNPGs with hepatic paraganglioma are exceedingly rare.

**Case presentation:**

We report a 59-year-old male patient with a 40-year history of an enlarged mass at the right side of the neck and two months of epigastric discomfort. Neck physical examination revealed a 6 × 6 cm, ovoid, firm mass on the right side of the neck. A pre-contrast computed tomography (CT) scan of the head and neck revealed bilateral heterogeneous soft tissue masses at the bifurcation of the carotid artery with indistinct border, the size of which was 2.4 cm × 2.6 cm on the left and 5.4 cm × 4.3 cm on the right. The lesions were intensely and heterogeneously enhanced with the internal and external carotid arteries surrounded and pushed anteriorly after contrast administration. Magnetic resonance imaging (MRI) showed a hyperintense signal on T2 weighted images compared to the surrounding muscle tissue and an intense contrast enhancement on T1 weighted images. Digital subtraction angiography (DSA) exhibited a highly vascularized masses that occupied and deformed both sides of the carotid bifurcation. As for the hepatic mass, non-contrasted CT imaging of the upper abdomen showed a 6.1 cm × 5.5 cm × 5.8 cm low density mass in the liver with indistinct border. On late arterial phase, the mass showed slight enhancement with an enlarged hepatic artery pushed around the lesion. MR imaging of the lesion in the liver demonstrated low signal intensity on T1 weighted images but heterogeneous high signal intensity on T2 weighted images. On diffusion weighted images, the mass showed high signal intensity whereas low signal intensity was seen on the image of apparent diffusion coefficient (ADC). Moreover, the contrast-enhanced MRI showed that the lesion was intensely but heterogeneously enhanced.

**Conclusion:**

Multiple HNPGs with hepatic paraganglioma are exceedingly rare. Advanced medical imaging modalities such as ultrasound (US), CT, MR, DSA and ^123^I-metaiodobenzylguanidine (^123^I-MIBG) are helpful in the evaluation of the patients with PGs. Increased awareness of their concomitant occurrence and familiarity with their characteristic features are critical for clinicians and radiologists to avoid diagnostic and therapeutic pitfalls and to facilitate the early diagnosis.

## Background

Paragangliomas (PGs), representing 0.012 % of all tumors, are rare and mostly benign neoplasms that originate from the neuroendocrine tissue along the paravertebral axis and thus may develop at various body sites [1–3]. PGs in head and neck account for about 0.6 % of all head and neck tumors, and the most anatomically prevalent sites found within the head and neck region are the carotid body, jugular body, along the glossopharyngeal nerve and its tympanic branch, and the vagus nerve [4, 5]. Head and neck paragangliomas (HNPGs) may occur either sporadically or in the context of a hereditary familial tumor syndrome [6]. Multilocular presentations of HNPGs are observed in 10 to 20 % of sporadic cases and nearly 80 % of hereditary patients [7]. However, PGs are barely multiple in origin or seen in multiple organs. Malignant HNPGs are known to metastasize but typically invade locally although there are very few case reports showing metastatic spread of primary HNPGs to the liver. To the best of our knowledge, the existence of multiple PGs in head and neck and the liver are extremely rare, which should be defined as malignant PGs according to the 2004 World Health Organization classification [2, 8, 9]. Unfortunately, no molecular or histologic markers exist to predict if a primary PG has metastatic potential [1, 2, 8].

Although Stoeckli et al. [10] had reported that not all HNPGs are highly vascularized, most of these masses harbor characteristic findings on radiologic imaging, appearing as well-circumscribed, strongly enhancing masses [4, 11]. However, preoperative diagnosis of hepatic PGs remains a big challenge because hepatic PGs are often confused with fibrolamellar hepatocellular carcinoma [12]. Advanced medical imaging technologies such as ultrasound (US), computed tomography (CT), magnetic resonance (MR), digital subtraction angiography (DSA) and ^123^I-metaiodobenzylguanidine (^123^I-MIBG) may be useful in the evaluation of the patients with PGs. To date, there are very few literatures describing the radiologic features of hepatic PGs. Here we report a case of multiple HNPGs with hepatic PGs and make a short review of the literature related to the radiologic features and therapeutic strategies of the rarely occurring multiple tumors.

## Case presentation

A 59-year-old man presented with a 40-year history of an enlarged mass at the right side of the neck and two months of epigastric discomfort. He denied fatigue, flushing, heat or cold intolerance, headaches. There was no abnormality of his familial history related to cancer or inherited diseases. Neck physical examination revealed a 6 × 6 cm, ovoid, firm mass on the right side of the neck. Laboratory evaluation revealed γ-glutamyltransferase 135 (normal range: 10–60 U/L), lactate dehydrogenase 104 (normal range: 109–245 U/L), creatine kinase 14 (normal range: 38–174 U/L), C-reactive protein 147 (normal range: 0–8 mg/L), D-dimer 12.23 (upper reference limit: 0.55 mg/L). The plasma levels of tumor markers alpha fetal protein (AFP) and CA199 were within normal limits.

**Fig. 1:**
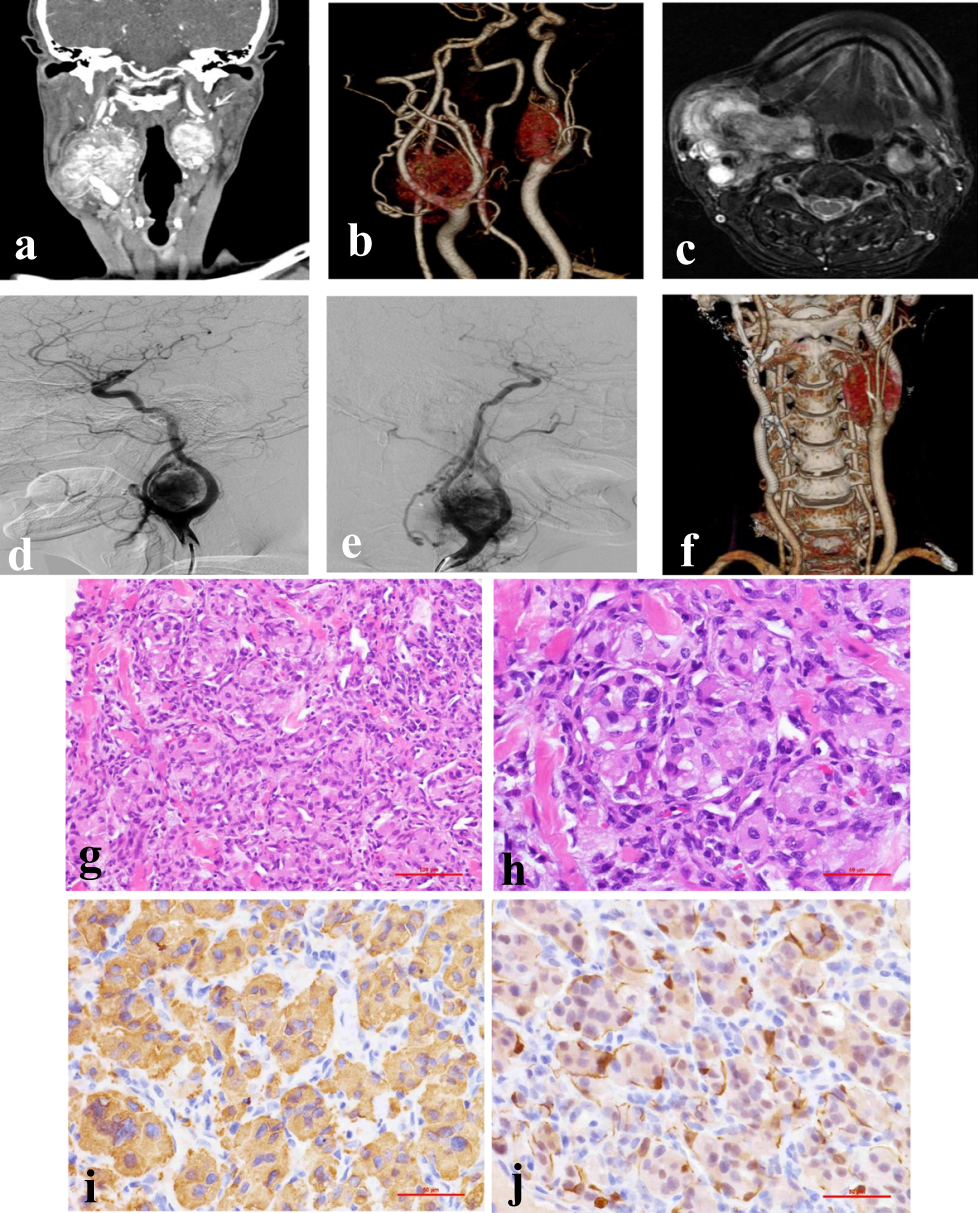
Radiologic, histologic and immunophenotypic features of HNPGs in the patient. **a** Coronal contrast enhanced multiple-planner reconstruction (MPR) image for a better visualization of the relationship between the masses and the carotid artery. Bilateral masses were detected at the bifurcation of both sides of the carotid artery, showing intense but heterogeneous enhancement with the internal and external carotid arteries surrounded and pushed anteriorly. **b** Computed tomographic angiography (CTA) image showing the tumor vascularity. **c** The axial gadolinium-enhanced T1-weighted image showing intense enhancement on the masses. **d**, **e** Digital subtraction angiography (DSA) showing highly vascularized masses occupying and deforming the bifurcation of both the right (**d**) and left (**e**) side of the carotid artery. **f** The postoperative CTA image showing the right mass excised and stent implatation in bilateral internal carotid arteries. **g**, **h** Haematoxylin and eosin staining of the HNPG specimen showing the nested (Zellballen) pattern of neoplastic cells and their neuroendocrine appearance (g: magnification, × 200; h: magnification, × 400). **i**, **j** Immunohistochemical staining for synaptophysin (**i**) and S-100 (**j**) highlighting the chief and sustentacular cells, respectively (magnification, × 400)

A pre-contrast CT scan of the head and neck revealed bilateral heterogeneous soft tissue density masses at the bifurcation of the carotid artery with indistinct border, the size of which was 2.4 cm × 2.6 cm on the left and 5.4 cm × 4.3 cm on the right. After contrast administration, the lesions were intensely and heterogeneously enhanced with the internal and external carotid arteries surrounded and pushed anteriorly (Fig. 1a-b). MRI showed a hyperintense signal on T2 weighted images compared to the surrounding muscle tissue and an intense contrast enhancement on T1 weighted images (Fig. 1c). DSA exhibited a highly vascularized masses that occupied and deformed both sides of the carotid bifurcation (Fig. 1d-e). The cardiovascular surgeon classified bilateral masses as type II in Shamblin classification [11, 13] and advised embolization and excision of the right mass, which was performed without complications. Furthermore, bilateral internal carotid arteries had undertaken angioplasty and stenting, considering that bilateral masses had shown the vascular invasion. The postoperative computed tomographic angiography (CTA) imaging confirmed the scope of operation (Fig.1f). Histopathological examination of the surgical specimen demonstrated that all masses were entirely covered with fibrous capsules, and its architectural pattern resembled “zellballen” (Fig. 1g-h). The size of the tumor cells varied and the nuclei of the tumor cells were round or oval with some tumor cells having large nuclei or multiple nuclei. Given the histological findings, immunohistochemical staining of neuroendocrine markers was performed to evaluate for possible differentiation. Synaptophysin was used to identify the chief cells of neuroendocrine origin whereas S-100 stained the sustentacular cells. The neoplastic cells were strongly immunoreactive for synaptophysin (Fig. 1i) and outlined by the supporting sustentacular framework as stained by S-100 protein (Fig. 1j).

**Fig. 2:**
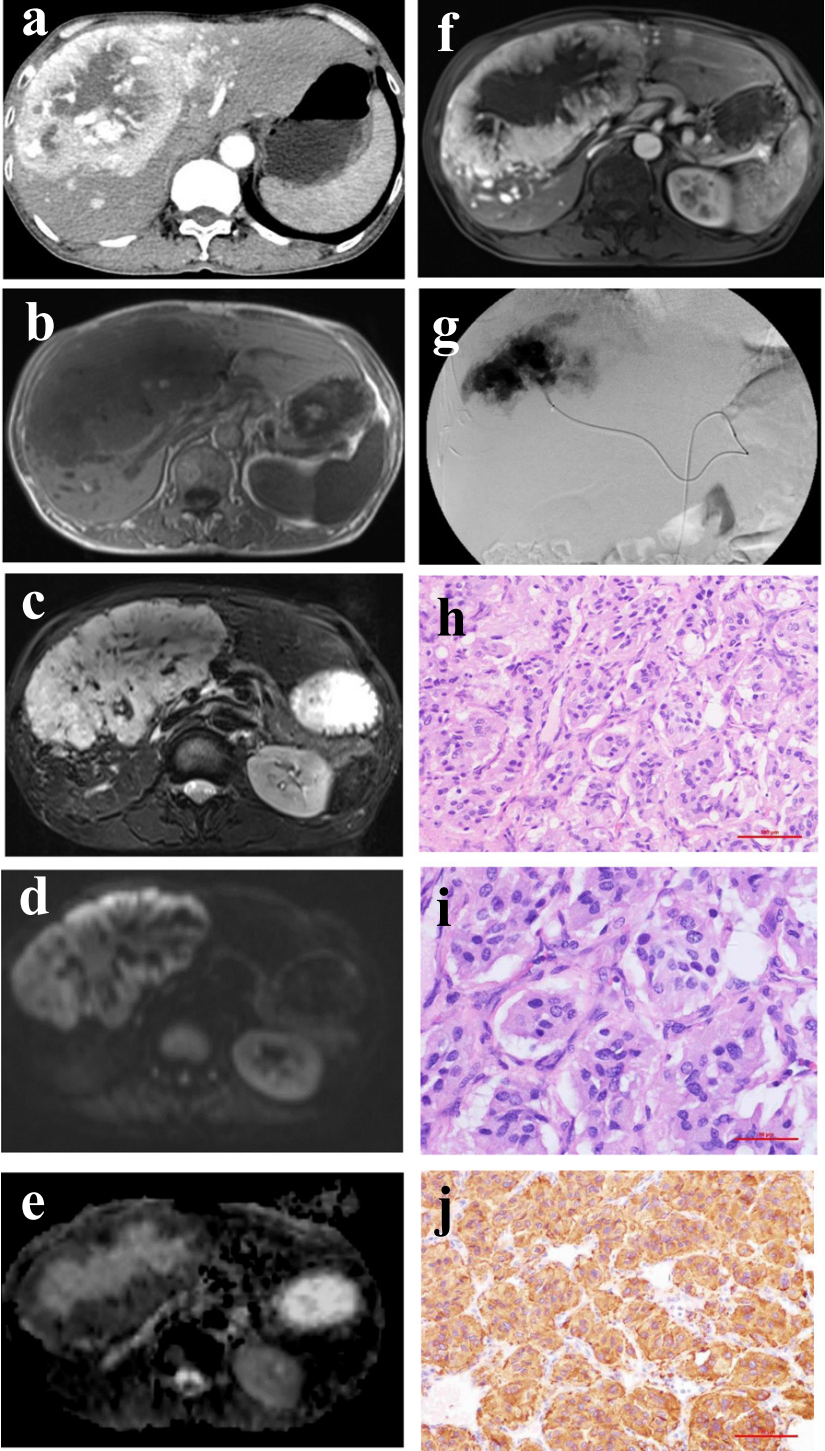
Radiologic, histologic and immunophenotypic features of hepatic PGs in the patient. **a** Late arterial phase image showing the slightly enhanced mass. **b** T1-weighted image exhibiting low signal intensity of the mass compared to the liver tissue. **c** T2 weighted image showing heterogeneous high signal intensity of the mass compared to the liver tissue. **d**, **e** Diffusion weighted image demonstrating high signal intensity of the solid component in the mass (**d**), whereas low signal intensity was seen on the image of ADC (**e**). **f** The contrast-enhanced MRI shows that the mass was heterogeneously and dramatically enhanced. **g** DSA showing that the hepatic mass was highly vascularized. **h**, **i** Haematoxylin and eosin staining of the hepatic PG specimen showing the nested (Zellballen) pattern of neoplastic cells and their neuroendocrine appearance (h: magnification, × 200; i: magnification, × 400). **j** Immunohistochemical staining for expression of synaptophysin (magnification, × 200)

Non-contrasted CT imaging of the upper abdomen showed a 6.1 cm × 5.5 cm × 5.8 cm low density mass in the liver with indistinct border. On late arterial phase, the mass showed slightly enhanced with an enlarged hepatic artery pushed around the lesion (Fig. 2a). MR imaging of the lesion in the liver demonstrated low signal intensity on T1 weighted images (Fig. 2b) but heterogeneous high signal intensity on T2 weighted images compared to the liver tissue (Fig. 2c). On diffusion weighted images, the solid component of the mass showed high signal intensity whereas low signal intensity was seen on the image of apparent diffusion coefficient (ADC) (Fig. 2d-e). Moreover, the contrast-enhanced MRI showed the lesion was intensely but heterogeneously enhanced (Fig. 2f). With the hepatic mass suspected as malignant tumor, transcatheter hepatic arterial chemoembolization was performed, which also confirmed the hepatic mass as a highly vascularized tumor (Fig. 2g). Three months later, a hemihepatectomy was performed to resect the mass lesion successfully. Histopathological and immunohistochemical study revealed that the mass contained a similar “Zellballen” pattern of tumor cells (Fig. 2h-i) and was immunoreactive for synaptophysin (Fig. 2j).

The patient recovered with uneventful course after surgical removal of the tumors and was followed annually for 8 years without clinical and radiological signs of recurrence.

## Discussion

Paragangliomas are considered to be benign tumors that arise from paraganglia [14, 15]. They may occur along the paraganglia’s pathway of embryologic migration from the skull base to the pelvic floor [5]. The incidence of head and neck PGs has been reported in a range of 1:30,000 to 1:100,000, comprising only 0.6 % of all head and neck tumors. Even rarer is multiple HNPGs with concurrent hepatic PG. To date, most hepatic PGs reported in the literature are considered as liver metastasis from extra-adrenal PGs since extra-adrenal PGs are associated with a relatively high rate of malignancy, ranging from 14 to 50 % [16]. Malignant HNPGs could spread both hematogenously and lymphatically with the most common sites of distant metastases being bone, liver, and lungs. Metastatic carotid body paraganglioma (CBPG) involved in the liver was reported to be as few as 10 cases [9]. In our case, the fact that the patient had a long history of prior neck mass and the hepatic lesion showed paraganglioma also points toward the probability that the hepatic PGs may be metastases of HNPGs. However, we cannot exclude a possibility that PGs both in the neck and in the liver developed separately and all were primary neoplasms. Although rare, CBPG with other PGs in the neck and thyroid has been documented [15, 17].

Due to the highly vascular nature of most HNPGs, an open or closed biopsy should not be attempted [4, 18]. The traditional gold standard for non-invasive diagnosis of HNPGs is DSA. However, other imaging modalities such as US, CT, MRI and ^123^I-MIBG are also necessary to establish the diagnosis [13, 18, 19]. In regards to the differentiation between HNPGs and other masses in the neck, schwannoma and lymphadenopathy should be taken into consideration. Due to the lack of high vascularity, the enhancement of schwannomas and lymphadenopathy are less than HNPGs [4]. Moreover, schwannoma often makes the internal and external carotid arteries displacing rather than splaying, which is a characteristic finding to differentiate HNPGs from schwannomas [15]. With respect to hepatic PGs, the diagnosis of this entity usually depends on pathology after resection or biopsy. There is no report in the literature regarding utility of advanced imaging techniques for diagnosis of hepatic PGs. The differentiation between hepatic PGs and fibrolamellar hepatocellular carcinomas (HCCs) are really difficult since hepatic PGs can show significantly enhanced on hepatic arterial phase as fibrolamellar HCCs do. In this case report, CT imaging revealed that the solid components of hepatic PGs were significantly enhanced while the necrotic elements displayed a non-enhancement. MRI on the hepatic PGs demonstrated low signal intensity on T1 weighted images and heterogeneous high signal intensity on T2 weighted images compared to the normal liver tissue. On gadolinium T1-weighted images, the solid components enhanced markedly and heterogeneously whereas the necrotic elements did not. Interestingly, hepatic PG in our case demonstrated signal void of vessels in tumors on T2-weighted images but lacking of central scar in tumors which is characteristic in fibrolamellar HCCs and probably a key point for the differentiation. Histologically, hepatic PG often resemble extra-adrenal PGs at other sites, which may lead to misdiagnosis of it as other common types of hepatic neoplasms. Thus, the accurate diagnosis may further rely on the immunohistochemical staining on tumor or tissue-specific markers.

The current treatment options for HNPGs include surgical resection, radiation therapy, permanent embolization or a combination of those modalities [20]. Surgical resection has been considered a standard of treatment for HNPGs and should be offered to patients unless the risk of surgery outweighs its potential benefits [3]. In our present case, bilateral HNPGs had showed partially surrounding the vessels, which made radical resection of both masses in the carotid bifurcation challenging. Taken all factors into consideration, embolization of the right mass and following excision will reduce intraoperative hemorrhage. Furthermore, bilateral internal carotid arteries had undertaken angioplasty and stenting, which may play an important role in preventing invasion. As for multiple HNPGs with hepatic PGs, because of the rarity clinical trials are not feasible and comparison of treatment outcome from very few patients reported is not sensible. As a result, management decisions for such patients are based on logical assumptions on the characteristics of this slow-growing pattern of the tumor and the theoretic benefits of the different treatment modalities [5]. When paragangliomas have metastasized, surgical eradication of the tumor and its regional confined lymph nodes would offer the best chance of cure. In our case, both hepatic mass and the regional lymph nodes had been completely resected. Although surgical outcomes for PGs are most favorable, post-operational and long-term follow-up with CT or MRI is highly recommended.

## Conclusions

In conclusion, multiple HNPGs with hepatic paraganglioma are exceedingly rare. Advanced medical imaging modalities such as US, CT, MR, DSA and ^123^I-MIBG are helpful in the evaluation of the patients with PGs. Increased awareness of their concomitant occurrence and familiarity with their characteristic features are critical for clinicians and radiologists to avoid diagnostic and therapeutic pitfalls and to facilitate the early diagnosis.

## Consent

Written informed consent was obtained from the patient for publication of this Case report and any accompanying images. A copy of the written consents is available for review by the Editor of this journal.

## Abbreviations

PGs: Paraganliomas
HNPGs: Head and neck paragangliomas
CBPG: Carotid body paraganglioma
CT: Computed tomography
MR: Magnetic resonance imaging
US: Ultrasound
DSA: Digital subtraction angiography
^123^I-MIBG: ^123^I-metaiodobenzylguanidine
HCC: Hepatocellular carcinomas
CTA: Computed tomographic angiography
MPR: Multiple-planner reconstruction
ADC: Apparent diffusion coefficient
